# Ancient orphan crop joins modern era: gene-based SNP discovery and mapping in lentil

**DOI:** 10.1186/1471-2164-14-192

**Published:** 2013-03-18

**Authors:** Andrew G Sharpe, Larissa Ramsay, Lacey-Anne Sanderson, Michael J Fedoruk, Wayne E Clarke, Rong Li, Sateesh Kagale, Perumal Vijayan, Albert Vandenberg, Kirstin E Bett

**Affiliations:** 1 National Research Council Canada, 110 Gymnasium Place, Saskatoon, SK, S7N 0W9, Canada; 2 Department of Plant Sciences, University of Saskatchewan, 51 Campus Dr., Saskatoon, SK, S7N 5A8, Canada; 3 Agriculture and Agri-Food Canada, Saskatoon Research Centre, 107 Science Pl., Saskatoon, SK, S7N 0X2, Canada

## Abstract

**Background:**

The genus *Lens* comprises a range of closely related species within the galegoid clade of the Papilionoideae family. The clade includes other important crops (e.g. chickpea and pea) as well as a sequenced model legume (*Medicago truncatula*). Lentil is a global food crop increasing in importance in the Indian sub-continent and elsewhere due to its nutritional value and quick cooking time. Despite this importance there has been a dearth of genetic and genomic resources for the crop and this has limited the application of marker-assisted selection strategies in breeding.

**Results:**

We describe here the development of a deep and diverse transcriptome resource for lentil using next generation sequencing technology. The generation of data in multiple cultivated (*L. culinaris*) and wild (*L. ervoides*) genotypes together with the utilization of a bioinformatics workflow enabled the identification of a large collection of SNPs and the subsequent development of a genotyping platform that was used to establish the first comprehensive genetic map of the *L. culinaris* genome. Extensive collinearity with *M. truncatula* was evident on the basis of sequence homology between mapped markers and the model genome and large translocations and inversions relative to *M. truncatula* were identified. An estimate for the time divergence of *L. culinaris* from *L. ervoides* and of both from *M. truncatula* was also calculated.

**Conclusions:**

The availability of the genomic and derived molecular marker resources presented here will help change lentil breeding strategies and lead to increased genetic gain in the future.

## Background

Lentil (*Lens culinaris* Medik.) is the world’s fifth largest pulse crop [1] with annual production of 3-4 Mt. It is produced in over 70 countries and is consumed in more than 120 countries. The crop was domesticated in southwest Asia and has been produced for several millennia [2]. Lentil is considered an orphan crop from a genomic perspective [3] primarily because of the relatively small production base imposed by climatic limitations to adaptation, the limited research investment in sequencing imposed by technology costs and the relatively large size (~4 Gbp; [4]) of the genome. Lentils are, however, a good source of protein, carbohydrates, micronutrients and vitamins for human nutrition, and their small size and flat shape make them relatively quick cooking and easily decorticated compared to most other grain legumes. This may account for increasing global per capita consumption over the past 50 years [5]. Lentil provides economic and biological benefits in cereal-based cropping systems through nitrogen fixation and crop rotation. In northern temperate, Mediterranean and sub-tropical savannah agricultural ecosystems, the lentil crop plays an important role in food and nutritional security on a global scale.

Until recently, molecular markers for use in lentil genetic mapping have been limited to anonymous markers such as RAPDs, ISSRs and AFLPs [6-11] and limited numbers of simple sequence repeat markers (SSRs; [12]). Phan et al. [13] added several intron-targeted amplified polymorphic (ITAP) gene-based markers to the collection of mapped loci. In the past five years, as the cost of genomic technologies declined, and as bioinformatic bridges were developed to access genomic information of fully sequenced model legumes like *Medicago truncatula*, concerted efforts were initiated to develop genetic marker technology platforms for lentil. These technologies will be of great benefit to future genetic improvement efforts and to future understanding of the relationships between the cultivated lentil and its wild relatives. These benefits have already been demonstrated to a limited extent by Alo et al. [14] who used *Medicago* EST sequences to develop primers that amplified conserved sequences in *Lens* spp. for diversity analysis. Application of second-generation sequencing technologies to lentil has also been reported by Kaur et al. [15], using 454 shotgun sequencing of cDNA samples from six different lentil genotypes to assemble unigenes and identify gene-based SSRs.

Recent advances in the development of genetic marker technologies have mainly been based on the availability of large numbers of single nucleotide polymorphisms (SNPs) between different genotypes of targeted organisms. The drive to implement single nucleotide variations as markers has come from the arena of human genetics where large numbers of SNPs have been identified in recent years [16]. The implementation of SNP markers in plant species in particular has been more limited, primarily because of the lack of available sequenced reference genomes. The identification of large numbers of SNPs for crops would allow the generation of dense linkage maps enabling applications such as high resolution QTL analysis, fine-mapping and map-based gene cloning [17,18]. The prospect of identifying markers very tightly linked to genes controlling traits of interest would also facilitate routine cost effective marker-assisted selection [19,20].

SNP marker technology is characterized by a large portfolio of genotyping methodologies that range from single-plex assays for interrogating individual loci to highly parallel multiplex assays, which can be used in various applications. For example, single-plex assays can be used for routine marker-assisted selection of thousands of individual plants for a targeted trait [21] while parallel assays can be used for the generation of high density genetic maps or association mapping studies [22]. Single-plex SNP assays that have been used in legume species include cleaved amplified target loci cleaved with restriction enzymes (CAPs; e.g. [23]), single strand confirmation polymorphism (SSCP; e.g. [24]), allele specific PCR amplification (Taqman: e.g. [25] and KASP: e.g. [26]). Multiplexed SNP assays include formats that accommodate either moderate levels of multiplexing such as that offered by mass spectrometry on the Sequenom MassArray platform [27] (Sequenom Inc., San Diego, CA) to the highly parallel allele specific PCR amplified assays of the GoldenGate and Infinium SNP arrays developed by Illumina (Illumina Inc., San Diego, CA) that are well established for a number of different crops including barley [28], maize [29] and soybean [22].

In this paper we describe the development of a collection of 3′-cDNA 454 reads derived from multiple *Lens* species and genotypes, the identification and characterization of SNPs from this data, the selection of a subset of SNPs for the development of a 1,536 SNP Illumina GoldenGate array and the use of the array to generate a comprehensive linkage map for a *L. culinaris* genetic mapping population.

## Results and discussion

### 454 Sequencing and SNP discovery

In order to identify a breadth of nucleotide diversity across the genic regions of the lentil genome a selection of nine *L. culinaris* genotypes and two *L. ervoides* wild accessions were selected for targeted 3′-cDNA transcript profiling using 454 pyrosequencing technology (Table 1). One genotype (CDC Redberry) was pre-selected as the reference genotype in order to generate approximately triple the amount of 454 sequence data (1.03 × 10^6^ reads) compared to the other genotypes and thus enable the development of a reference *de novo* assembly. This process produced a base assembly consisting of 50,146 contigs which were then filtered based on duplication, overlap and size to produce a reference assembly of 27,921 contigs. The relatively large number of identified contigs can be explained by the targeted 3′ nature of the 454 transcript sequence data that enables a high read depth representation of the 3′ ends of genes. This avoids the issue of limited read depth across the entire gene space that is commonly observed with shotgun cDNA 454 sequence data, and that consequently limits the ability to identify robust sequence variation across multiple genotypes. The high average read depth for all the different genotypes demonstrates the success of this strategy (Table 1).

**Table 1:** High quality SNP discovery results in ten lentil lines against the reference genotype CDC Redberry

**Line**	**Type**	**Origin**^**1**^	**Adaptation**^**2**^	**Total 454 reads**	**Reference assembly**	**Contigs with SNPs**	**Total SNPs**	**Average read depth**
CDC Redberry	Small red	Canada	NT	1,034,231	27,921	N/A	N/A	N/A
CDC Robin	Small red	Canada	NT	394,707	N/A	2177	4727	12
964A-46	Large green	Canada	NT	370,621	N/A	1967	4138	10
Eston	Small green	Turkey	NT	410,494	N/A	1240	2690	17
PI 320937	Black	Germany	NT	419,342	N/A	2003	4276	11
LC8602303T	Small red	USA	MED	366,006	N/A	2762	6371	13
ILL 5588	Small red	Jordan	MED	217,539	N/A	1684	3740	8
CDC Milestone	Small green	Canada	NT	592,086	N/A	1569	3390	31
ILL 8006	Small red	Bangladesh	STS	523,958	N/A	4304	9797	15
L01-827A	*L. ervoides*	^**3**^	MED	218,657	N/A	3264	10,793	10
IG 72815	*L. ervoides*	ICARDA	MED	370,513	N/A	5847	19,946	10
Total				4,918,154		11,050^**4**^	44,879^**4**^	

All lines are *L. culinaris* except the last two, which are *L. ervoides*.

^**1.**^ Origin indicates where line was originally collected or developed through hybridization and selection.

^**2.**^ NT – northern temperate; MED – Mediterranean; STS – sub-tropical savannah.

^**3.**^ Single plant selection from IG 72847.

^**4.**^ Total non-redundant set of contigs and SNPs detected across all genotypes, respectively.

In contrast, an assembly of such shotgun 454 data from six different lentil genotypes in a previous study used approximately the same amount of 454 data (1.36 × 10^6^ reads), but identified only 15,354 contigs [15]. Thus, the main advantage of developing large numbers of high quality reference 3′ contigs for SNP discovery is that in theory it will enable a more robust identification of common transcripts and nucleotide variants when 3′-cDNA data from other genotypes is assembled against them. The larger representation of untranslated regions (primarily 3′-UTRs) of genes in the data also offers the prospect of identifying higher levels of sequence variability when compared to data containing a higher proportion of protein coding sequences, as well as a offering a better resolution of reads derived from multiple members of closely related gene families.

The development of reference assemblies of the 3′-cDNA 454 data from the nine other *L. culinaris* genotypes and two *L. ervoides* genotypes using a custom bioinformatics pipeline (Additional file 1), confirmed our assumption by identifying large numbers of polymorphic contigs and SNPs for each genotype when compared against the CDC Redberry reference. A set of 11,050 non-redundant polymorphic contigs (41% of all contigs) were identified from this analysis, containing a total of 44,879 non redundant SNPs (Table 1). These SNPs comprised 27,198 transitions (61%) and 17,681 transversions (39%) closely reflecting the nucleotide conversions seen in other plants [30]. The average allele frequency of the identified SNPs was 0.31 for all lines and 0.21 for the *L. culinaris* genotypes alone. This difference reflects that the wild accessions are derived from a secondary or perhaps tertiary genepool [2,31] and thus a much higher degree of sequence diversity between the two species is expected. The level of nucleotide diversity within the *L. culinaris* genotypes (Table 1) appears to follow the adapted origin of the genotypes where overall lower levels are observed between genotypes adapted to a northern temperate climate and higher levels are observed between these genotypes and others adapted to either a Mediterranean (e.g. ILL 5588) or a sub-tropical savannah environment (e.g. ILL 8006). ILL 8006 has twice as many unique SNPs relative to CDC Redberry compared to the next most diverse line, ILL 5588. Developed in Bangladesh and released as Barimasur-4 [32], ILL 8006 is derived from a cross between ILL 5888 (a selection from a local land race in Bangladesh) and ILL 5782 selected for tolerance to rust and stemphylium blight. It is well adapted to the local growing regions of Bangladesh but does not perform well in northern temperate climates due to photoperiod sensitivity (A. Vandenberg, Univ. of Saskatchewan, unpublished observations). This makes it more difficult to use in northern temperate breeding programs as a source of diversity. The two genotypes that are adapted to a Mediterranean climate had twice as many unique SNPs relative to CDC Redberry compared to the northern temperate genotypes.

### Sequence characterization

An *in silico* analysis of non-redundant contigs for the presence of microsatellite repeats revealed 529 contigs (4.7%) that contained such a repeat and represent a resource for potential genetic marker development. This will complement identified repeats from other initiatives using similar sequencing strategies in this crop [15].

Comparative analysis of all 27,921 reference contigs to the sequenced annotated model legume genomes of *M. truncatula* and soybean [33,34] revealed 17,550 (62.9%) contigs that possessed closely related orthologous genes in the model legume genomes (*M. truncatula* = 12,821 independent contigs; *Glycine max* = 4,729 independent contigs. Additional file 2). The 12,821 contigs with orthologous counterparts in *M. truncatula* represents 30.4% of the total annotated genes (42,118) in this species and represents the limitation of transcript profiling in only a small set of plant tissues. It also reflects the 3′ nature of the contigs that just represent the 3′-UTR of genes in a significant proportion of cases, limiting the effectiveness of identifying orthologous genes. The same analysis was carried out for just the 11,050 (39.6%) polymorphic, non-redundant contigs and identified 8,696 (78.6%) contigs with orthologous copies in the related model legume genomes, and accounted for 36,893 (82.2%) of the total set of non-redundant SNPs. The classification of the gene models identified for contigs from the total set and contigs from just the polymorphic contigs was also generated according to the available and well established Arabidopsis gene ontology (GOSlim) categories for cellular, molecular function and biological processes (Additional files 2 and 3). This revealed a broad range of classes of genes for each set and established that 3′ genic polymorphism is represented well across all classes of genes. The range of genes is also similar to the GOSlim classes identified and already described by the shotgun approach adopted by Kaur et al. [15] and is unsurprising considering the range of plant tissues utilized in the two studies is similar. A comparison of the GO slim between the total set of contigs, all polymorphic contigs, only those polymorphic between cultivated genotypes and only those polymorphic between wild genotypes (Additional file 3) revealed a very similar profile of gene classes in each case with no evidence of any selective pressure. An overall trend in a higher proportion of the different classes being represented in the polymorphic contigs is likely explained by an overall longer length of these contigs compared to the total set of contigs (see below).

That there were 10,371 (37.1%) total and 2,354 (21.4%) polymorphic lentil contigs with no orthologous loci in either model is not surprising considering the 3′ nature of the contigs; however, it is also possible that a lack of an orthologous gene is due to a high degree of divergence between orthlogous genes in the different species, the presence of lentil specific genes, or an incomplete representation of genes in the current model genome assemblies. The higher percentage of total contigs with no orthologous counterparts compared to polymorphic contigs can also be explained by a lower average contig length of the total set (451 bp) compared to the polymorphic set (666 bp), thus reducing the amount sequence available for comparative analysis of this nature.

Annotation of the gene models in *M. truncatula* and soybean for polymorphic contigs also enabled SNPs to be classified as putative protein coding (exonic) or non-coding (3′-UTR) in nature. This revealed 17,748 SNPs as potentially exonic in nature and 5,039 potentially in 3′-UTRs. The classification of SNPs as exonic allows the potential for the impact of the observed variation on the codon reading frame and an estimate of time of divergence between *L. culinaris* and *L. ervoides* based upon the observed synonymous substitution rate (Ks; Additional file 4). Synonymous changes that were only observed between *L. culinaris* and *L. ervoides* (mode Ks 0.07) enabled an estimate of the time of divergence at 677,000 years ago. Synonymous changes observed between *L. culinaris* and *M. truncatula* led to an estimate of divergence between these two species at 38 Million Years Ago (MYA) which is similar to existing estimates between *Lens* and *Medicago*[35]. In total 6,830 polymorphic contigs contained 21,511 SNPs in exons where reading frame and codon could be determined across all *L. culinaris* and *L. ervoides* genotypes. Of these, 8,963 SNPs encoded a synonymous codon change and 12,548 SNPs a non-synonymous codon change. For the latter class 7,714 SNPs encoded a different amino acid property (i.e., basic vs. acidic), 4,834 SNPs resulted in a non-synonymous codon change with the same properties and 147 SNPs that would result in a truncated protein due to the presence of a stop codon. Interestingly, only a few less synonymous (8,958) and non-synonymous (12,545) changes were observed when only comparing amongst the *L. culinaris* genotypes, revealing, at least in the coding regions of this subset of genes, very little difference in observed polymorphism between *L. culinaris* and *L. ervoides*. The large number of the same changes in both species relative to *Medicago* further reflects the significant time of divergence from a common ancestor as well as the very recent divergence of *L. culinaris* from *L. ervoides*. Since the number of genotypes from each species in this study is relatively small we cannot comment on the occurrence of discrete artificial selection sweeps during the domestication and adaptation of *L. culinaris* as observed in other domesticated species [36], or the potential for multiple origins of domestication for this species [14]; however, the prospect of the availability of a whole genome sequence for *L. culinaris* in the relatively near future, as well as the availability of increasing amounts of sequence data from multiple *L. culinaris* genotypes and other *Lens* species, will help to further elucidate the nature of the domestication process for this crop.

### Development of a SNP genotyping platform

**Figure 1:**
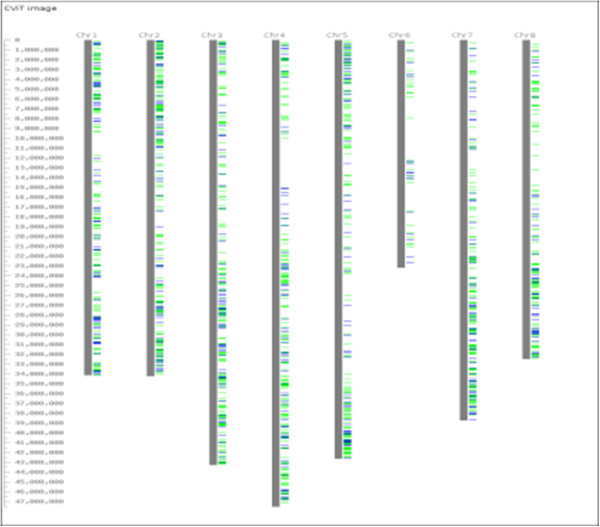
**Based on *in silico *mapping of corresponding *Medicago truncatula *homologues, polymorphic *Lens *contigs span the *Medicago truncatula *genome (CviT;**[37]**).** The 1,536 selected for a GoldenGate OPA are indicated in blue while the rest are indicated in green.

Based on *in silico* mapping of *M. truncatula* homologues, the 8,060 polymorphic contigs, including the 1,536 selected for SNP array development, are evenly distributed across the genome (Figure 1) suggesting we have sampled the gene space across the lentil genome using the targeted transcriptome profiling strategy.

KASP assays were initially designed around 28 SNPs within unique contigs for in-house validation in the 11 genotypes (Additional file 2). Four of the assays failed: one (LcC00012p343) was designed to bridge an exon/intron splice site based on *M. truncatula* and therefore was not expected to be reliable. The other three failures were possibly due to technical issues and/or erroneous *M. truncatula* annotation. One contig (00093) had no predicted exon/intron boundaries and it worked in some, but not all, genotypes. Of the remaining, all worked in most of the 11 genotypes and none gave erroneous results across all genotypes. There were several instances where there was a mismatch but this could be due to the inherent heterogeneous nature of lentil cultivars.

To further test the SNPs, an additional 124 were screened on the SNP discovery lines using the KBioscience service facility in the UK. When screened on the parental lines, none failed and only 4.2% did not match the 454 SNP allele call (Additional file 2). Between the LR-18 parents, 56 were polymorphic and segregated in the mapping population so were used for mapping.

Selection of the 1,536 SNPs for the GoldenGate array was based on them being reported as polymorphic between at least two of the eight cultivated lentil genotypes and the reference line CDC Redberry. Eight of the 11 genotypes on the initial 454 sequencing panel were genotyped using this GoldenGate array. Validation of these results was carried out by comparing them with the 454 base calls. Of the 751 data points examined, 97% matched the expected genotype (Additional file 2) demonstrating the robustness of the SNP discovery strategy employed in this study. These genotypes represent parents of existing mapping populations so the data can be queried to determine if there are sufficient numbers of polymorphic loci to warrant genotyping the segregating lines with the Lc1536 GoldenGate OPA or, if there are too few polymorphic SNPs, whether it would be more economical to design single-SNP assays for genotyping. The array could also be used to genotype diverse germplasm to assess genetic diversity and plan future genetic studies.

### Linkage mapping and comparative analysis

**Figure 2:**
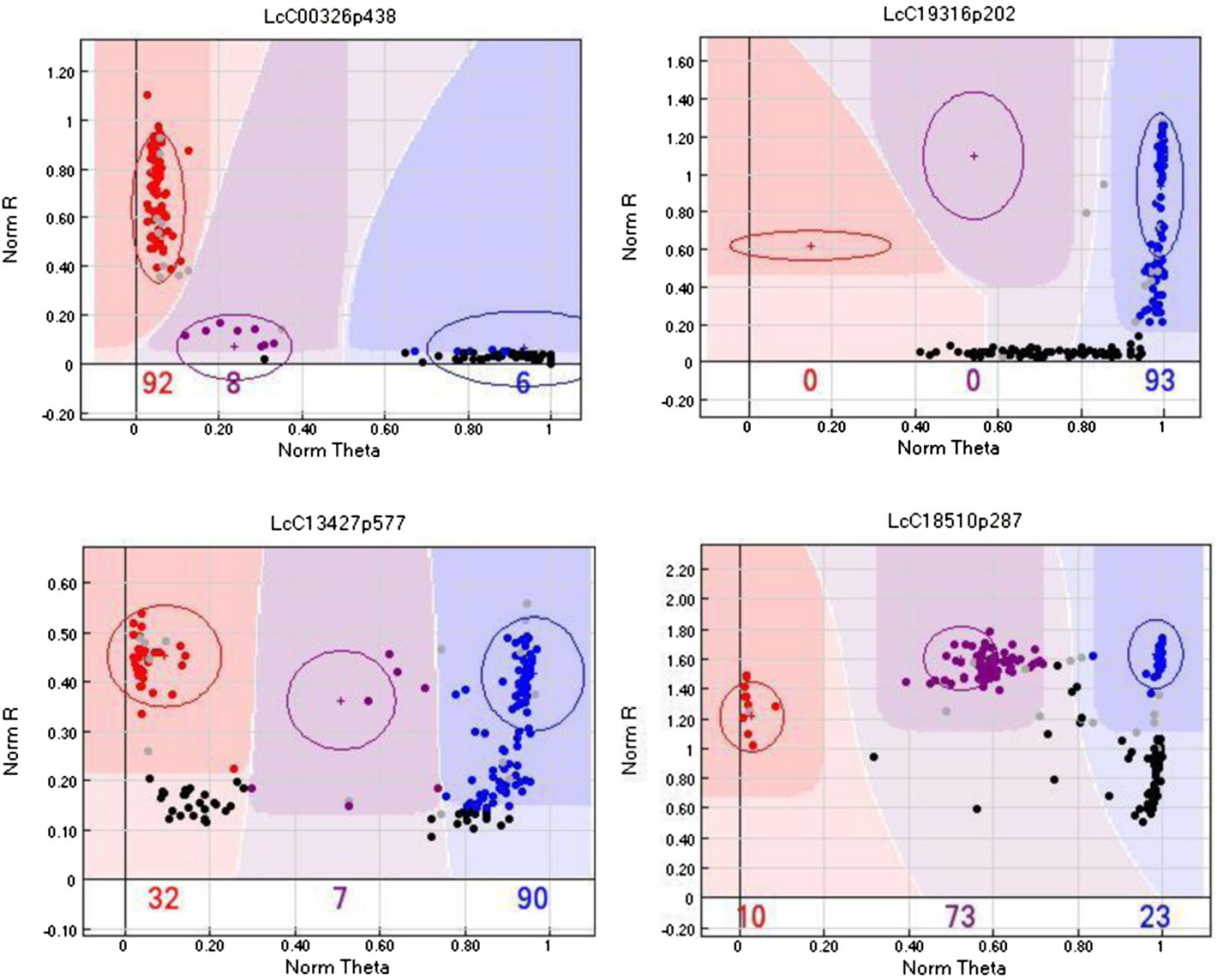
Examples of SNP loci that segregated in a dominant fashion (top two plots) or like duplicated genes (bottom two plots) based on GoldenGate array output as visualized in Genome Studio version 2010.3 (Illumina Inc.).

The LR-18 population was genotyped with the Lc1536 GoldenGate OPA. Almost one third (484) of the loci were polymorphic and could be used for mapping purposes. This was in line with what was expected based on the 454 SNP allele calls. Of the remaining 1,052 assays, 848 (81%) were monomorphic in this population, 154 (14.6%) failed completely, 20 (2%) had a pattern where one of the alleles failed to amplify but the other did (dominant markers; Figure 2 top) and 30 (2.9%) had patterns that appeared to represent gene duplications and could not be readily scored (Figure 2 bottom). Further investigation of this last class of assays revealed irregularities with 19 contigs that were indicative of the presence of sequence duplication. The presence of segregation patterns that suggest duplicate genes is not surprising since all legumes share an ancient and defining duplication event and 20% of genes are tandemly duplicated in *M. truncatula*[33]. The bioinformatics screening we employed tried to eliminate duplicates being used for the SNP assay design but the nature of the 3′ data and inherent complications of *de novo* assembly approach for the reference likely limited its effectiveness. Most of the dominant markers segregated in a fashion that allowed them to be scored by classifying the null allele according to the parent that had the same genotype. These markers were included in the mapping data and all mapped to one or other of the linkage groups.

Of the polymorphic SNPs assayed using KASP assays, 19 were also represented on the Lc1536 array resulting in some contigs having multiple SNP genotypes. This was invaluable in confirming the data from both types of genotyping were the same and also allowed missing data to be filled in from other SNPs in the contig. For mapping purposes, a haplotype based on the consensus genotype was generated for each individual contig.

**Figure 3:**
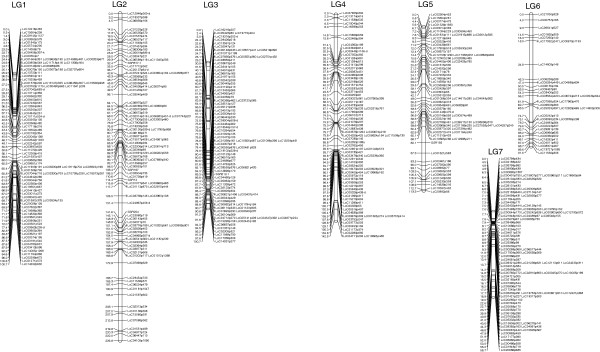
**Genetic linkage map of *Lens culinaris *RIL mapping population LR-18 (CDC Robin × 964a-46) based on gene-based SNP (LcC#) and SSR (SSR#) genotyping.** The seven linkage groups likely represent the seven chromosomes of lentil. Cumulative distances are in cM.

**Figure 4:**
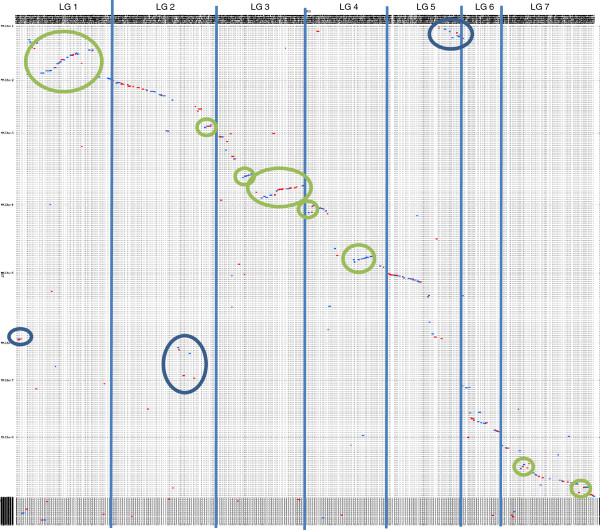
**Dot plot representing correspondences between lentil linkage groups 1 through 7 (top) and *Medicago truncatula *chromosomes 1 through 8 (left side) based on alignment and visualization using MUMmer and NUCmer software [**[38]**].** Major translocations are circled in blue and major inversions in green.

There were six SSRs and 537 contigs that could be mapped in LR-18 (Figure 3). The map has seven linkage groups that likely represent the seven chromosomes in lentil. The linkage groups range from 58 cM to 226 cM and together they cover 834.7 cM. There are a few large gaps but this is likely the result of mapping with gene-based markers in a genome that consists largely of repetitive elements and regions of low polymorphism in an intraspecific population derived from an adapted by adapted cross. As there is considerable collinearity between lentil and *M. truncatula* across these regions (Figure 4), we are comfortable with these gaps in this initial map. Mapping in additional populations will enable us to add more markers to these regions, since many of the gaps are due to a lack of polymorphism between the parents of LR-18, and build a consensus map that fills in these gaps. This mapping population was chosen for the first map due to the diverse genetic backgrounds of the parents: one is a small red lentil and the other a large green lentil and the population segregates for multiple traits for which this map will be used in the future to elucidate genetic control of these traits. This map will also form the basis for future efforts in the genetic anchoring and assembly of scaffolds and contigs derived from on-going genome sequencing initiatives (Sharpe, Ramsay and Bett, unpublished).

This is the first intraspecific gene-based map of the lentil genome that can be resolved down to the same number of linkage groups as there are chromosomes. As such, it will form the basis for future mapping efforts for the lentil genome leading to the identification of markers for traits of importance in lentil breeding. The availability of this map along with easy-to-use SNP markers linked to traits of interest will allow lentil breeders to finally incorporate marker-assisted selection into their breeding programs. The availability of such markers and a high through-put genotyping system will lead to improved selection efficiency and increased genetic gain in lentil breeding programs.

The lentil linkage groups have been numbered to match the corresponding *M. truncatula* chromosomes as best as possible (Figure 4). In general there are only a few translocations and a number of inversions that distinguish lentil synteny from that of *M. truncatula*. Most notably, Mt chromosome 6 seems to be collinear with the middle of lentil linkage group 2, which is otherwise collinear with Mt chromosome 2. A similar break in synteny occurs when *M. truncatula* is compared to Lotus [39], *M. sativa* and *Pisum sativum*[40]. There is also a translocation involving the ends of lentil linkage groups 1 and 5 that are translocated with respect to *M. truncatula*. Finally, there are major inversions relative to *M. truncatula* in lentil linkage groups 1, 3 and 4 together with smaller ones within most other linkage groups. The density of markers used for comparative analysis in this study is approximately six times higher than that used in previous analyses [13] and the extensive blocks of collinearity we observe between lentil and *M. truncatula* provides an inherent confidence in this genetic map for lentil.

All data presented in this study are available on our publically searchable database KnowPulse: Pulse Crop Breeding and Genetics (http://knowpulse2.usask.ca/portal). Specifically, the assembled 454 sequence contigs are available for search by name or through the BLAST tool provided by KnowPulse. This allows researchers to find contigs with high sequence similarity to their gene of interest and thus narrow the set of polymorphic loci to those most likely to be linked to their trait of interest. The identified sequence loci known to be polymorphic between at least two of the lines are listed on each contig with their location and any known allele calls. These loci can be inspected by contig or as an independent set in the genotype listing tool where potential markers are shown as rows in a table with the known allele calls in user selected germplasm comprising the columns. This genotype listing can be narrowed to only those potential markers expected to be polymorphic (i.e. different allele calls for a given marker) between any two user-selected genotypes allowing researchers to inspect the total known differences between the two. The loci included on the Lc1536 GoldenGate OPA and full assay details, including primers for any KASP assays designed, are presented via this portal informing researchers of already developed markers and providing all the information necessary for the integration of these markers directly into a research project or breeding program.

Having a set of gene-based markers with demonstrable collinearity with a model legume will be of use in future efforts to identify candidate genes for traits of interest. The database can be searched for orthologues of candidate genes from other species and SNPs within any of the lentil contigs identified, including those not represented on the array. As the database of SNP allele calls expands, lines and populations likely to be segregating for these SNPs can be identified and used in phenotyping experiments to help confirm their association with traits of interest.

## Conclusions

The targeted transcriptome profiling methodology and generated 454 sequence data described here represents a significant addition to existing genomics resources for *Lens* species. It has enabled a large number of SNPs across a diverse range of genes to be identified in a diverse range of domesticated *L. culinaris* genotypes and also wild *L. ervoides* accessions. The collection provides a wealth of nucleotide variation that will be used by researchers and breeders for genetic analysis and breeding programs for years to come. It enabled the generation of an Illumina GoldenGate SNP array and the production of the first comprehensive SNP-based genetic linkage map for *L. culinaris*. The map, together with the use of the array in other genetic mapping or association mapping populations, will provide the basis from which trait-based mapping can be carried out in the future. The developed genetic map also enabled a detailed comparative analysis with the model *M. truncatula* genome and the observed conserved synteny with this and other model legume species will ultimately facilitate the identification of candidate genes that regulate key traits in this important food crop.

## Methods

### Plant material

Nine *L. culinaris* and two *L. ervoides* genotypes, sourced from a wide geographical range, were used for SNP discovery (Table 1). A single plant of each genotype was selfed to produce sufficient seed to plant to generate tissues for library construction.

CDC Redberry [41] was used as the reference genome since it was the first small red lentil cultivar to combine resistance to both ascochyta blight and anthracnose, excellent lodging tolerance and high yield. It has a diverse genetic origin and has been a key genotype in the Canadian red lentil breeding program since 2005. It was the recurrent parent for backcrossing imidazolinone tolerance to create the cultivar CDC Maxim which now accounts for more than 75% of red lentil production in North America.

CDC Robin and 964a-46 are the parents of the recombinant inbred line (RIL) population LR-18 that was used for genetic mapping. CDC Robin is a small red cultivar released in 1999 [42] and 964a-46 is a large green breeding line from the lentil breeding program at the Crop Development Centre (CDC), University of Saskatchewan. The RILs were bulked at F_8_ and a set of 147 lines has been grown and phenotyped extensively for the past five years. Lines were at F_12_ or higher. DNA was extracted from freeze-dried leaf tissue collected from at least five plants of each genotype using a modified CTAB method [43].

### 3′anchored cDNA library construction and sequencing

Five kinds of tissue samples were collected individually: (1) 2-week old leaf, (2) stem before flowering, (3) 1-week-old etiolated seedling, (4) mixed flower stages, and (5) developing seed at mixed stages. Total RNA from leaves was extracted using the RNeasy Plant Mini Kit (Qiagen) including on-column DNase digestion. Total RNA from other tissues was extracted using the CTAB method described by Meisel et al. [44] and then cleaned up using RNeasy Mini kits (Qiagen), including on-column DNase digestion. Two kinds of RNA samples were used for cDNA synthesis for library construction. For the first CDC Redberry library, equal amounts of the total RNA from each tissue were mixed and directly used for cDNA synthesis. For all other libraries, including a second CDC Redberry library, equal amounts of the total RNA from each tissue was mixed first, then further purified using the RiboMinus Plant Kit for RNA-Seq (Invitrogen), and then used for cDNA synthesis.

3′-anchored cDNA libraries for 454 sequencing were prepared based on a protocol described in Eveland et al. [45] and modified to incorporate *Aci*I as the restriction enzyme used to generate 3′ cDNA fragments of the optimal size range for amplification during 454 Titanium chemistry sequencing [46]. The use of *Aci*I as the appropriate enzyme was determined by *in silico* digestion of ESTs collected from chickpea (Sharpe and Cram, NRC Saskatoon, unpublished). First-strand cDNA was generated from ~5 ug of total RNA or RiboMinus RNA (derived from ~8 ug total RNA) using the ArrayScript kit (Ambion) according to manufacturer’s instructions. The synthesis was carried out using a biotinylated oligo(dT) fused to the 454 B-adapter primer, 5′ –biotin-CCTATCCCCTGTGTGCCTTGGCAGTCTCAG(T)_12_VN-3′. First-strand cDNA was then treated with 2 ul of RNase Cocktail Enzyme Mix (Ambion) for 30 min at 37°C. Double-strand cDNA was synthesized immediately by addition of 30 U of *E. coli* DNA Polymerase I and 1 U of *E. coli* RNase H (Fermentas Canada Inc.) to the first-strand synthesis reaction, following the manufacturer’s suggested protocol for second-strand cDNA synthesis.

The double stranded cDNA product was purified using MiniElute PCR Purification Kits (Qiagen Inc.), and then digested with the restriction enzyme *Aci*I (New England Biolabs) to create 2-base 5′CG overhangs for 454 A-adaptor ligation. The A-adapter was prepared by annealing 1μl each of top and bottom strand oligos (100 pmol/μl; top strand, 5′-CCATCTCATCCCTGCGTGTCTCCGACTCAGCAT-3′; bottom strand, 5′-CGATGCTGAGTCGGAGACACGCAGGGATGA-3′), 1 μl 10X Annealing Buffer (10 mM Tris-HCl, pH 8, 150 mM MgCl_2_, 150 mM NaCl, 1 mM spermadine), and 7μl dH_2_O for a total volume of 10 μl. The annealing mixture was heated at 55°C in a heating block for 5 min followed by slow cooling to room temp of the block together with samples. 30 μl dH_2_O was then added to achieve a final concentration of 2.5 pmol/μl for A-adaptor stock.

The *Aci*I-digested cDNA was treated with Agencourt AMPure Beads (Beckman Coulter Inc.) to remove smaller fragments (< 250 bp). The 3′-fragments of cDNA were recovered using DynBeads M-270 streptavidin (Invitrogen) and then ligated with A-adaptor. Unligated adaptors were removed by washing beads twice with 1× B&W Buffer (5 mM Tris-HCl, pH 7.5, 0.5 mM EDTA, 1 M NaCl, 0.01% Tween-20), and then fill-in reactions for repairing the nicks were done by using *Bst* DNA Polymerase, Large Fragment (New England Biolabs). To isolate the single-stranded AB adapted library, the immobilized beads were washed twice with 1× B&W Buffer and twice with Molecular Biology Grade water (Sigma). The desired 5′-A-cDNA-B-3′ template strand was eluted with 125 mM NaOH, neutralized, and concentrated on a column from the MinElute PCR Purification Kit (Qiagen Inc.).

The quantity and quality of the resultant single-stranded DNA library was assessed using a Q-PCR strategy [47] by using Platinum SYBR Green qPCR SuperMix UDG (Invitrogen). Molecules per microliter of the library were calculated using a 578-bp DNA standard while amplification through emPCR primers: forward primer, 5′-CCATCTCATCCCTGCGTGTC-3′; reverse primer, 5′-CCTATCCCCTGTGTGCCTTG-3′. The qPCR products were also checked on a 1.2% agarose gel to assess the quality of the libraries.

Roche 454 Titanium sequencing of the titrated single strand DNA libraries was carried out following the procedure described by Margulies et al. [48] with modifications for the Titanium chemistry as described in protocols supplied by the manufacturer (Roche, Laval, Quebec).

### Sequence assembly and analysis and SSR analysis

NGen (DNAStar) software was used for *de novo* assembly of the CDC Redberry 454 reads. Parameters used for the *de novo* assembly included: Min/Match Percent = 90; Max454 Sequence Length = 600; Repeat Handling On; and Expected Coverage = 20. Processing was carried out on a Dell R910, 2 × 2.40 GHz, 48GB RAM Windows 64Bit server. Following assembly, repeat class contigs were removed as well as any remaining contigs of <200bp. Sequence data from the other genotypes was then assembled against the CDC Redberry *de novo* reference assembly using NGen (DNAStar). To ensure removal of the 454 key sequence from reads, 10bp were trimmed from the 5′ ends and screened for contamination using the vector/adapter file. Adapter screening was used to remove the wobble primer, the adapter, and any poly-A tail. Contaminant filtering was implemented for a set of seven ribosomal sequences that were found to remove the majority of contaminant reads.

The identification of candidate SSRs was carried out using the software QDD [49] in the contigs generated from CDC Redberry.

The *L. culinaris* contigs, their *L. ervoides* orthologues and the corresponding *M. truncatula* genes were aligned using ClustalW and those without indels (628 contigs in total) were used to estimate evolutionary divergence time. Ks values were obtained with the codeml program in the PAML program suite [50]. The estimate of divergence was derived from the mode Ks of binned values, based on a mutation rate of 5.17× 10^-3^ substitutions/synonymous site/Myr for the legume lineage [34].

### SNP reporting

Seqman Pro (DNAStar) was used to identify SNPs relative to CDC Redberry. Individual reports were parsed into spreadsheet format to compare using a custom Perl pipeline. Only transitions and transversions were reported; indels were ignored as the nature of the pyrosequencing technique makes them less robust. The final spreadsheet report indicates if the SNP is the same as the reference; the alternate allele, or if there is no sequence data at that position (Additional file 2). All low confidence SNPs (represented in <80% or <3 reads) were identified and reported as being below thresholds if found in the same position as confident SNPs.

The 454 contigs were mapped against *M. truncatula* and soybean genomes using GMAP [51] with the cross-species parameter. Flanking sequence length and gene annotation information was extracted from the sequence mapping output through custom Perl scripting.

GO categorization was assigned based on the high-quality annotation information available for *Arabidopsis thaliana*. Contigs were compared against a database of TAIR10 CDS sequences, and the highest-scoring hit used to assign Arabidopsis GOslim gene ontology terms (since any one contig can be assigned to multiple GOslim terms, the total percentage in each category could exceed 100%).

### Validation

A set of 28 SNPs were chosen for initial validation (Additional file 2); two were chosen to test the effect of interfering SNPs and splice sites. Two allele specific forward primers and a common reverse primer were designed for use in fluorescence based competitive allele-specific PCR assays (KASP; KBioscience, Hoddeston, UK). DNA from the 11 individuals from the SNP discovery panel was assayed using these primers and KASP reaction mix (version 3 chemistry; KBioscience) following manufacturer’s instructions. PCR amplification was carried out in a StepOnePlus™ Real-Time PCR System (Applied Biosystems) and end-product fluorescence readings were analysed using StepOne Software v2.1 (Applied Biosystems).

Sequence data for contigs containing a further 150 SNPs were sent to KBioscience (Hoddesdon, UK) for design of additional KASP assays. Of these, it was possible to design assays for 124 SNPs and genotyping was carried out at KBioscience on 144 LR-18 RILs and on 10 individuals from the SNP discovery panel. The resulting data were visually inspected for allele calling errors using SNPviewer software (KBioscience) and corrected when deemed necessary.

### Illumina GoldenGate OPA design

All SNPs in the collection (44,942 amongst 11,061 contigs) were surveyed and only those that revealed polymorphism amongst *L. culinaris* genotypes and revealed polymorphism between at least two *L. culinaris* genotypes and the CDC Redberry reference were kept for further interrogation. Sequence data for the contigs surrounding the SNPs was checked for the number of base pairs to the end of the contig, to the next SNP, or to the closest splice site. Those with less than 60 bp in this flanking region were eliminated since they cannot be used for Illumina GoldenGate arrays (Illumina Inc., San Diego, CA). All remaining candidate SNPs were submitted to Illumina for assay design and a total of 7,229 SNPs were returned with design scores greater than 0.4; preferential selection was given to ones scoring above 0.6 as recommended by Illumina.

To reduce the number of SNPs to 1,536 for final array design, only one SNP per contig or one per predicted SNP haplotype was selected. Only one SNP assay for duplicated contigs or single assay for each duplicate was selected and the final selection was based on even spread across the genome by inference of distribution from contig homology to *M. truncatula* gene models. This final set of 1,536 was submitted to Illumina for design and synthesis of the Lc1536 OPA.

### Mapping

A total of 144 RILs from the LR-18 population were genotyped using the Lc1536 OPA and a standard Illumina GoldenGate assay protocol (http://www.illumina.com/technology/goldengate_genotyping_assay.ilmn). Products generated by this assay were read with an Illumina HiScan (Illumina Inc., San Diego, CA) and the resulting data were clustered for allele calling using GenomeStudio software version 2010.3 (Illumina Inc., San Diego, CA). Allele calls were visually inspected for errors in automatic allele calling and corrected where deemed necessary. Any calls that were not clearly one allele or the other were reported as missing data to avoid errors. Five individuals were removed before mapping due to too many missing data points, leaving 139 individuals for map development.

Lentil SSR markers from Hamwieh et al. [12] were screened on the parents of LR-18 and those that were polymorphic were used to genotype the whole population. To enable marker analysis using an ABI 3130× Genetic Analyzer (Applied Biosystems, Carlsbad, CA), forward primers were synthesized with an additional M13 universal primer sequence on the 5′ end. These forward primers were used in three-primer PCR amplifications along with the respective reverse primers and a 5′ fluorescently labeled (HEX, NED or FAM) M13 universal primer sequence. PCR reactions were conducted as described by Schuelke [52] with slight modification to the reaction conditions as described in Ubayasena et al. [53]. Product sizes were determined using GeneScan™ 500 ROX Size Standard (Applied Biosystems).

The LR-18 allele calls from all genotyping were exported for analysis and mapping using JoinMap 4.0 [54]. Maximum likelihood mapping was used and LOD values of 6 or higher were used to group loci into linkage groups to form the map. Regression mapping was used to finalize the map order of each linkage group and genetic distances were determined using the Kosambi mapping function. Map order was verified visually by examining the raw genotypes and the linkage map was generated using MapChart [55].

Contigs containing SNPs present in the linkage map were processed through the NUCmer pipeline [38] and the results filtered for global alignment using length × identity weighted longest increasing subset. Dotplots were generated using MUMmer plot and further visualized by parsing NUCmer output with custom Perl scripts.

### Data availability

KnowPulse: Pulse Crop Breeding and Genetics (http://knowpulse2.usask.ca/portal) is a repository of legume genetic and genomic data. The assembled 454 sequencing data for the lentil reference line (CDC Redberry) is available with all polymorphic loci and associated markers (KASP and Illumina Golden Gate arrays) indicated. Researchers can use a BLAST interface to identify potential homologues of their gene of interest based on sequence similarity. This allows them to both find existing markers associated with their gene of interest, as well as export information on specific loci in a variety of formats to aid in marker design. A comparative GBrowse (Generic Model Organism Database (GMOD), http://gmod.org) with a *M. truncatula* genomic backbone graphically displays sequence similarity-based homology between legume species providing an alternative approach to finding candidate markers. For an overview of all data made available through this project, visit the project page (http://knowpulse2.usask.ca/portal/node/3214572).

Sequence data has been deposited in the NCBI Sequence Read Archive (SRA) under the study accession SRP019372.

## Competing interests

The authors would like to declare that they have no financial or non-financial competing interests in the publication of this manuscript.

## Author’ contributions

KEB, AGS and AV were the co-PIs on the projects that led to this manuscript; they conceived of the study, participated in its design and coordination and co-wrote the manuscript. AGS oversaw the 3′ cDNA library construction and sequencing portions of the work. KEB oversaw the genotyping and mapping portions of the work. AV selected the germplasm used for sequencing and developed the LR-18 mapping population. MJF carried out the genetic mapping of LR-18. LAS prepared the data for dissemination through KnowPulse and the additional files and provided bioinformatic support throughout the project. RL provided input in terms of RNA extraction and established the adapted form of the 3′ cDNA profiling methodology. PV participated in the design and coordination of the study. SK provided methodologies for estimates of the time of divergence between species. LR provided bioinformatics support from SNP selection through data analysis and prepared several figures. WEC developed the SNP discovery pipeline. All authors read and approved the final manuscript.

## Supplementary Material

Additional file 1 Bioinformatics workflow for lentil SNP discovery and selection for the Illumina genotyping platform. Click here for file

Additional file 2 Contig and SNP data including lists of all contigs, all SNPs, gene homology with Medicago or soybean, SNPs selected for KASP and GoldenGate assays, and the results of these assays on a panel of 11 lines. Click here for file

Additional file 3 Classification of the gene models according to Arabidopsis GO Slim categories. Click here for file

Additional file 4 **Ks plots representing the frequencies of substitutions per synonymous site for aligned othologous contigs between *M. truncatula *and *L. culinaris *(a) and between *L. culinaris *and *L. ervoides *(b).** Click here for file
