# Supplementary material for: Ancient orphan crop joins modern era: gene-based SNP discovery and mapping in lentil
Source: BMC Genomics. 2013 Mar 18;14:192. doi: 10.1186/1471-2164-14-192 (PMC3635939; doi:10.1186/1471-2164-14-192)
Supplement: Additional file 1 — Bioinformatics workflow for lentil SNP discovery and selection for the Illumina genotyping platform. [file 1471-2164-14-192-S1.docx]

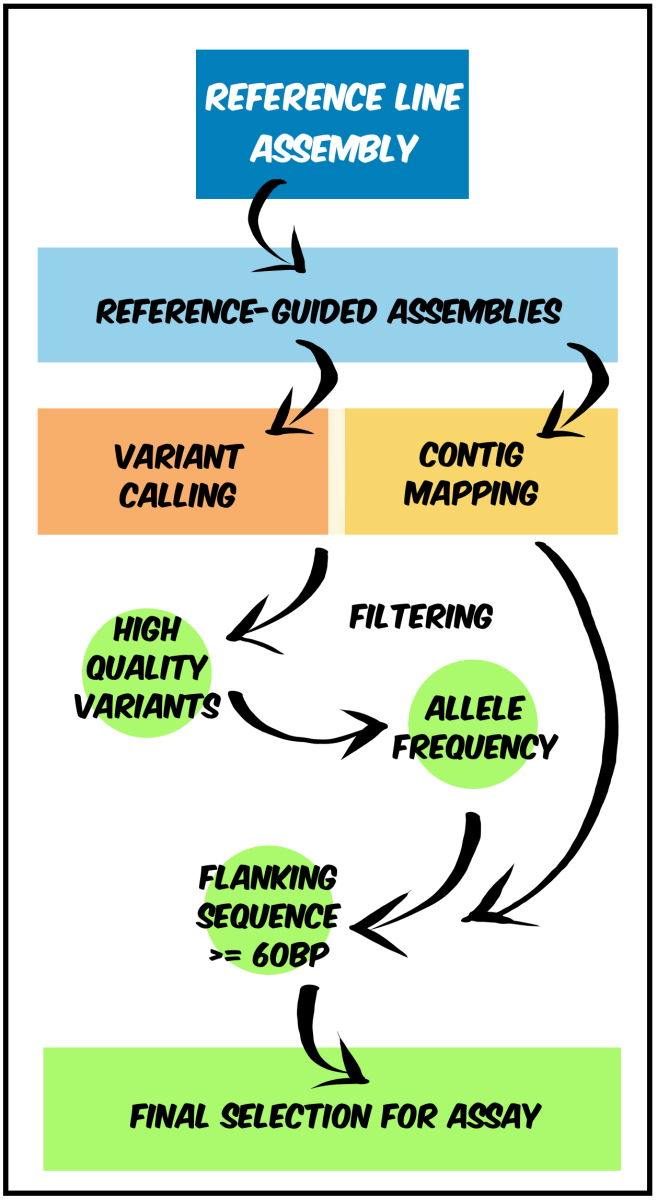


**Additional File 1.** Bioinformatics workflow for lentil SNP discovery and selection for the Illumina genotyping platform. SNP discovery was reliant on a reference guided approach and mapping of contigs to Medicago gene models. The exact description of individual steps in the workflow is described in the Materials and Methods. All output from the different steps of the workflow is represented within KnowPulse (<http://knowpulse2.usask.ca/portal> ).
