# Supplementary figures and images for: Ancient orphan crop joins modern era: gene-based SNP discovery and mapping in lentil
Source: BMC Genomics. 2013 Mar 18;14:192. doi: 10.1186/1471-2164-14-192 (PMC3635939; doi:10.1186/1471-2164-14-192)

## Slide 1
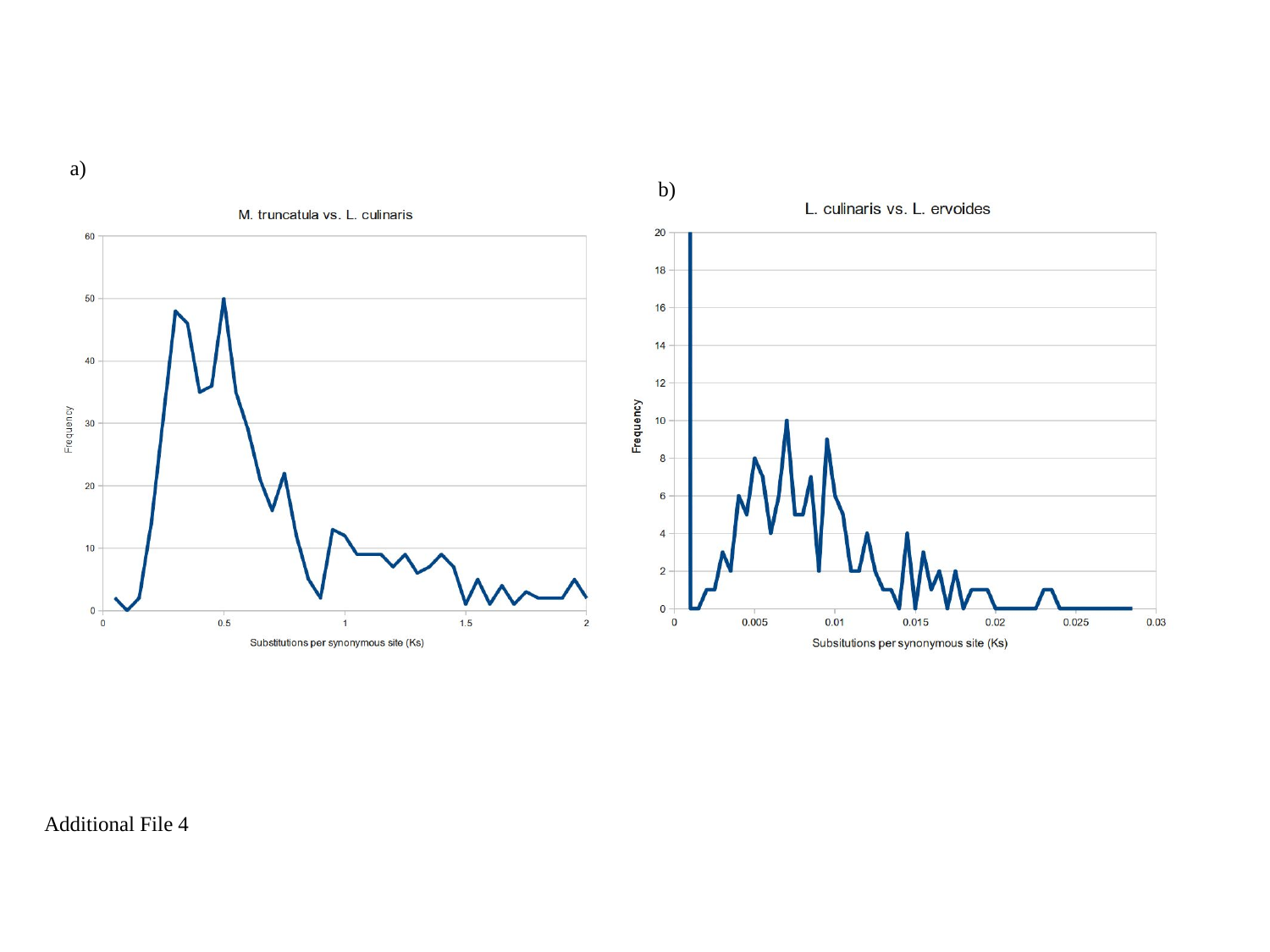

a)
b)
Additional File 4

Supplement: Additional file 4 — Ks plots representing the frequencies of substitutions per synonymous site for aligned othologous contigs between M. truncatula and L. culinaris (a) and between L. culinaris and L. ervoides (b). [file 1471-2164-14-192-S4.pptx]
